# Hepatitis C virus screening and treatment in Irish prisons from a governor and prison officer perspective - a qualitative exploration

**DOI:** 10.1186/s40352-018-0081-6

**Published:** 2018-12-19

**Authors:** D. Crowley, M. C. Van Hout, C. Murphy, E. Kelly, J. S. Lambert, W. Cullen

**Affiliations:** 1 Irish College of General Practitioners, Lincoln Place, Dublin, Ireland; 2 Public Health Institute, Liverpool John Moore’s University, Liverpool, UK; 3 Irish Prison Service, Mountjoy Prison, Dublin 7, Ireland; 4 Department of Infectious Diseases, School of Medicine, University College Dublin, Mater Misericordiae University Hospital, Dublin, Ireland; 5 School of Medicine, University College Dublin, Dublin, Ireland

**Keywords:** Hepatitis C virus, HCV, Prison, Prison officer, Governor, Screening, Treatment, Dual loyalty

## Abstract

**Background:**

Prisons are a key location to access Hepatitis C Virus (HCV) infected people who inject drugs (PWID). Prison health care structures are complex and optimising health care delivery to this high need, marginalised and underserved population remains challenging. Despite international guidelines recommending that prisons are a priority location for HCV screening and treatment levels of prisoner engagement in HCV care remain low. Competing priorities between security and healthcare is a key feature of prison health care. A collaborative approach to health care delivery in prisons can maximise the benefits for prisoners, staff and the wider community.

**Aim:**

To identify the barriers and enablers to HCV screening and treatment in Irish prisons and inform the implementation of a HCV screening program within the Irish Prison Services (IPS).

**Methods:**

Qualitative study using focus group methodology underpinned by grounded theory.

**Results:**

The following themes emerged from the analysis: priority of safety and security, staffing and resources, concerns about personal risk, lack of knowledge, concerns around confidentiality, prisoners’ fear of treatment and stigma, timing of screening, use of peer workers, in-reach hepatology and fibroscanning services. The primary role of prison security is to ensure the safety of staff and prisoners with a secondary but important supporting role in health care delivery. Maintaining adequate staffing levels and the provision of training and education were seen as priorities and impacted on prison officers’ fear for personal safety and risk of HCV transmission. Opt-out screening and peer support workers had high levels of support among participants.

**Conclusion:**

Upscaling HCV management in prisons requires an in-depth understanding of all barriers and facilitators to HCV screening and treatment. Engaging prison officers in the planning and delivery of health care initiatives is a key strategy to optimising the public health opportunity that prisons provides.

## Introduction

Hepatitis C Virus (HCV) infection is a leading cause of liver related morbidity and mortality across Europe. An estimated 14 million people are chronically infected with this blood borne virus in the European region and over 70,000 dying annually from HCV related liver cirrhosis and cancer (WHO 2017). Injecting drug use (IDU) is the major driver of the HCV epidemic in developed countries and now accounts for 80% of new infections in the European union (Degenhardt et al. 2017; WHO 2017).

The incidence and prevalence of HCV infection is much higher in people who inject drugs (PWID) than the general population (Degenhardt et al. 2017; Nelson et al. 2011). It is estimated that over 50% of PWID globally have chronic HCV infection (Nelson et al. 2011; WHO 2017). Across Europe the prevalence of anti-HCV among PWID ranges from 15%–84% with the average prevalence almost 50 times higher than the general population (WHO Europe 2015). Ongoing criminalisation of PWID ensures that they experience high rates of incarceration (56%–90% ever being incarcerated) and previous incarceration is associated frequently with HCV infection and increased injecting risk in the community (Cepeda et al. 2015; Jürgens et al. 2011). Recent prison release is also associated with heightened HCV transmission risk which is particular concern since the majority of prisoners globally (including Ireland) serve short prison sentences (Binswanger et al. 2007; Irish Penal Reform Trust 2018; Stone et al. 2017).

There are an estimated 3674 persons incarcerated across 15 institutions in the Republic of Ireland (ROI) on a daily basis (Irish Penal Reform Trust 2018). Irish prisoners are some of the most marginalised people in Irish society and despite having high rates of physical and psychiatric morbidity are underserved by traditional health services (Allwright et al. 2000; Drummond et al. 2014). They suffer high levels of poverty, social deprivation, homelessness, early school dropout, unemployment, illiteracy, mental illness and drug use (Barry et al. 2010; Drummond et al. 2014). Over half of Irish prisoners report a history of drug use and 43% a history of IDU (Drummond et al. 2014). An estimated 13% of Irish prisoners have been infected with HCV (Drummond et al. 2014). HCV prevalence varies across Irish prison facilities and is related to the number of PWID incarcerated at the location (Allwright 2000; Drummond et al. 2014; Long et al. 2001). National and international HCV strategies and treatment guidelines identify prisoners as a key population to screen and treat for HCV infection yet uptake within prisons remain low (Department of Health 2017; European Association for the Study of the Liver 2017; European Monitoring Centre for Drugs and Drugs Prevention 2016; Health Service Executive 2012; Martin et al. 2015).

The availability of once daily, easy to tolerate, highly effective pan-genotypic direct acting anti-virals (DAA), mobile elastography for liver disease assessment and less restrictive treatment guidelines has revolutionised HCV management (Crowley et al. 2017; European Association for the Study of the Liver 2017; Grebely et al. 2017). HCV infection is a curable and preventable disease and the World Health Organisation (WHO) have declared its elimination by 2030 a public health goal (WHO 2017). Despite this optimism many challenges remain to engage high-risk populations in HCV care. A number of studies have identified prisoner and nurse reported barriers to HCV management in prison settings including: concerns regarding stigma and discrimination, lack of information regarding screening procedures, fear of treatment, competing priorities and short prison sentence and inter-prison transfer (Khaw et al. 2007; Yap et al. 2014). Enablers to prison –based HCV care have also been identified and these include: dried blood spot testing, opt-out screening, in-reach hepatology, tele-medicine, nurse-lead clinics and peer support (Arora et al. 2007; Lloyd et al. 2013; Martin et al. 2013; Morris et al. 2017).

It is recognised that prison health care structures are complex and the relationship between health care workers and security staff can impact health care delivery (Fazel and Baillargeon 2011; Møller et al. 1936; WHO 2014). The concept of dual loyalty has been described in the literature which can create conflict between health care professionals and security staff (Pont et al. 2012). While the maintenance of security and safety is the first priority for prison governors and officers, they also play a crucial supportive role in prison health care (Ismail and de Viggiani 2018). Prison governors’ and officers’ views on health care delivery are rarely reported in the literature. We only found one study that explored prison officers’ views on HCV screening and treatment published in the literature to date (Jack et al. 2017). This study aims to explore prison governors’ and officers’ views on barriers and enablers to HCV screening and treatment which will inform the development of Irish public health strategy to increase HCV screening and treatment in prisoners nationally. It may also inform international approaches to prison -based HCV management and increase awareness and knowledge about the complex and important relationship that exists between security and health care staff in prison and how this can impact health care delivery.

## Methodology

Five focus groups were conducted among two grades of security staff: the prison governor (*n* = 3) and prison officer (*n* = 2). In the Irish prison system (IPS), the overall security and operational issues for each prison is managed by governors with prison officers responsible for delivery of front-line services. Prison officers report to governors. The governor component of the study is national in coverage and includes input form 13 of the 15 prisons in the ROI. Usually Governors have also worked as prison officers in the earlier part of their careers. For convenience and due to restricted access to other prison locations, the prison officer focus groups were confined to two Dublin prisons: Mountjoy male prison and the Dochas female prison. Many of the prison officer participants had worked in many different prison locations across the country and this experience is included in their contribution to the study. The focus group methodology was chosen to allow for the maximum number of participants to contribute, its acceptability in a real-life setting and to capture the richness, complexity and scope of the issues**.** Ethical approval was obtained from the Mater Ethics Committee as part of the Seek and Treat component of The European Hep Care Project and supported and endorsed by the IPS ethics group (Lambert et al. 2018).

Different recruitment strategies were used for different focus groups. For the governor focus groups, invitations were sent by email to all governors working in the IPS. The focus groups were organised on days and at times that coincided with national monthly meetings to maximise attendance and minimise disruption. The recruitment of prison officers was by open invitation, with times and dates of focus groups advertised on local noticeboards, the electronic staff communication system and word of mouth. Local staff groups were conducted on-site in communal private rooms deemed suitable by the research team. National groups were conducted off-site and the Irish Prison Service College (Stack House). Data collection took place during a three-month period in 2017. All participants were given a participant information sheet (PIS) containing information on the study and had opportunities to ask questions before giving their informed written consent to participate.

Following a review of the published literature on the topic, completion of a scoping review and consultation with the research group and national experts in the area a focus group guide was finalised. This guideline included a series of open ended questions covering the following areas; experience of prison healthcare delivery including HCV screening and treatment, barriers and enablers to prisoners’ engagement with prison-based HCV care, challenges related to incarceration and release, inter-prison variations in health care and the role of prison officers and peers in prison HCV management. Each group was moderated and observed by two researchers from the research team. Focus group interviews were recorded using an encrypted digital audio recorder, and observations of group dynamics and interactions were written in field notes during and after the event. After each focus group, audio recordings were transcribed using Microsoft word and were uploaded to NVIVO 11 for coding and thematic analysis. Interview data were coded as collected and the topic guides revised accordingly. A grounded theory approach informed both the collection and analysis of the data. The thematic coding was revised with the analysis of each focus group and analysis ceased when thematic saturation was achieved (agreed by researcher 1 and 2).

## Results

Five focus groups were conducted involving a total of 68 participants. Seven females were included in the study which is a lower representation that expected given that females represent 16% of prion officers and 30% of governors in the IPS. The five focus groups produced 7.5 h of narratives. The following themes emerged from the analysis: priority of safety and security, staffing and resources, concerns about personal risk, lack of knowledge, concerns around confidentiality, prisoners’ fear of treatment and stigma, timing of screening, use of peer workers, in-reach hepatology and fibroscanning services.

### Priority of safety and security

All focus groups included discussions about issues of security and safety in their prisons. While supportive and understanding of the benefits of prison health care their primary focus was to ensure the safety of both staff and inmates.

Local prison officers spoke of the increased security concerns related to protection of prisoners and how burgeoning gangland feuds and rival factions made their jobs very difficult. This created barriers to both HCV screening and treatment since it reduced face to face time with prisoners and medical staff because security staff are required to accompany prisoners to medical appointments.

*“Keeping our prisons secure is the first priority – that is where we start every day*”. (Governor)

*“Supporting healthcare is important but we need to ensure that staff are covering the landings first*”. (Governor)

*“In some areas (of the prison) have trouble getting around, takes half an hour, you can’t cut corners ...... if you turn your back they ‘d kill each other*” (Prison officer)

### Staffing and resources

Safety and security concerns were closely underpinned by lack of staff and resources. This was a persistent theme throughout all narratives. Lack of custodial staff impacted negatively on the delivery of health care.

“*I think it’s … it’s looking at how you maximise operations, .... the most efficient system in place and how to support that. Whether it’s more officers or nurses. There should be more supports in the prison and resources should be going into the prison rather than spending it on sending prisoners back and forth to the hospital*” (Governor)

*“There has to be a plan. Has to be enough staff. People who are trained”* (Prison officer)

Hospital visits outside the prison were viewed as an inefficient and resource heavy way of providing health care to prisoners. Minimising hospital visits by expanding in-reach services (bringing specialist medical services into the prison) were seen as facilitating prisoners’ access to health care including HCV care.

*“Anything that stops us going outside the main gate is good”*. (Governor)

### Concerns about personal risk

A recurring theme throughout the prison officer focus groups was concern for personal safety. This concern covered the areas of personal safety and risk of exposure to and acquisition of blood borne viruses including HCV. Prison officers described a work environment of increasing inter-prisoner violence and severity of assault often leading to open wounds and blood loss.

*“I worry about myself--------, with the increased violence actually………, we are exposed more and more to open wounds and stuff”* (Prison officer)

*“Our biggest one is blood to blood …. violence with blood to blood contact that is the main one we probably have here”.* (Governor)

*“Staff are still worried – they have families, children”.* (Prison officer)

### Lack of knowledge

Lack of knowledge was recognised as a major barrier to HCV screening and treatment. Participants identified the provision of education and training as a means of addressing this knowledge deficit. All grades of staff felt their lack knowledge in relation to the newer HCV treatment and the risks of transmission impacted on their ability to engage with prisoners on this issue.

*“Blood viruses are a big issue in our prisons ...staff awareness must be brought forward as well, they have to buy into it as well”.* (Governor)

*“Staff worry about HIV and HCV ... we need more education programs and training for staff but it also hard to release staff for training*”. (Governor)

Participants also identified the lack of knowledge among prisoners as a barrier to HCV treatment in particular the inaccurate information being circulated regarding the side effects of treatment which were historical and associated with interferon-based treatment.

*“…. a lot of prisoners don’t know what they have or have been tested even if they are at high risk”.* (Prison officer)

*“It’s patchy, the prisoner’s knowledge that we come across .... they don’t really know how to access treatment”. (Prison officer)*

### Concerns regarding confidentiality

Medical confidentiality was discussed in all groups and it was acknowledged to be a prisoner right and an essential tenet underpinning health care delivery.

“*Prisoners are concerned that we access records for things like DNA* ... *we (custodial staff) have no access what so ever to PHMS (electronic patient record)”* (Governor)

Front line prison officer participants had a much more nuanced discussion in relation to this topic with a majority reporting that lack of confidentiality was a barrier to HCV screening and treatment. Often breaches in confidentiality were inadvertent and were related to prisoners being called to attend certain clinics /doctors that were connected with HCV, addiction treatment or HIV care.

*“It is difficult at times- when prisoners are called to see the Hep (*in-reach hepatology*) nurse or the phy (*methadone*) doctor ... why else would they be seeing them”. (Prison officer)*

Prison officers reported that some patients treated with interferon-based therapies suffered significant side effects from treatment and these were so visible that both staff and fellow prisoners knew that they were having HCV treatment.

*“Guys who’ve lost a lot of weight, often very noticeable weight loss with previous treatments ……… common knowledge among staff and prisoners that they are on a course of Hep treatment*” (Prison officer)

A number of participants described examples of prisoners forgoing their privacy by openly discussing their health issues with both non-clinical staff and fellow prisoners.

*“Ah sure some of them don’t care who knows what -----, they would come up to you and say here medic can you get me an appointment” (Prison officer)*

A number of officers felt that if issues regarding confidentiality were addressed that more prisoners would approach prison officer to discuss HCV related concerns and that this might be a resource to educate prisoners on HCV related issues.

*“I feel that they would come to me if things were a bit more private” (Prison officer)*

### Prisoners’ fear of treatment and stigma

A number of participants identified fear of treatment as a barrier to prisoners engaging with health services. Fear of treatment was linked to: side effects of interferon treatment, liver biopsy and the concerns about stigma. Participants also identified that making screening routine or opt-out, had the potential to reduce stigma.

*“They won’t show up to the doctor because they’re scared”.* (Governor)

*“In terms of history and patient issues, patients think they’re going to get very sick on it (interferon), they think they’re going to end up in a heap”.* (Prison officer)

*“I hear them (prisoners) say “don’t get that liver biopsy”, they’re still afraid ... word of mouth is a big thing in the prison*”. (Prison officer)

*“Stigma – they have been dealing with that for years – fear of HIV -body suit the works*”. (Prison officer).

*“We need to reduce the stigma – making it routine is the way to go”.* (Governor)

### Time of screening

Both prison officer and governor participants favoured a structured and systematic approach to HCV screening. The committal period was identified by all groups as an opportune time to engage prisoners with health services and provide HCV screening. Security and health care staff are prioritised to cover the committal process which is standardised and involves the completion of an electronic nursing health assessment.

*“Committal is a good time to get them tested -----, it’s always staffed, in fact you should make it compulsory ----of course, unless they know their test result already*” (Prison officer)

*“Big benefit is that if you know he needs treatment, you can sort it straight away”.* (Governor)

*“Obviously the best time to do it is on committal, you don’t want to go to pick them up a few weeks later and it’s hard because the turnover is so big. You’d nearly be starting again”.* (Prison officer)

Many felt that screening should be compulsory on committal with small numbers believing that it should be voluntary.

“At the committal stage it should be compulsory”. (Prison officer)

*“It’d have to be voluntary”.* (Governor)

Some prison officers identified other time periods that might be suited to HCV screening. They described “down times” within the week where routine work was not scheduled. They also noted that health related programs provided during these times might have the added benefit of relieving boredom for prisoners

*“There are down times in here – Sunday after mass, prisoners are bored and would get involved” (Prison officer)*

### Peer workers

Participants in all focus groups agreed that trained peer workers had the potential to facilitate prisoner engagement with health services including HCV screening and treatment. A number of participants had engaged in the past with mass BBV and TB screening initiatives with peer workers from the Irish Red Cross. The narrative around peer workers included prisoners having more trust in their peer networks than “The System”.

*“Peer led education is better than say something coming from the top down. Let the guys at the ground work up.”* (Governor)

*“Peer to peer ……. .and it was very successful when we did it the last time ……. using the volunteers, like we did the last time, we got a 78 % buy in because of that*.” (Prison officer)

“*They see us may be pulling the wool but if it came from themselves ……. we’d be able to get over that hurdle”.* (Prison officer)

*“They wouldn’t come up and talk to you (prison officer). They’d be asking about getting a blood test to each other. They wouldn’t come to you”.* (Prison officer)

### In -reach hepatology and fibroscanning services

The availability of in-reach hepatology and mobile elastography were seen as enablers to prisoner engagement in HCV care. The cost –effectiveness and staff saving benefits of in-reach services were viewed by the governor focus groups as a major benefit. The reduction of risk associated with prisoners having to attend hospital services was also noted. In-reach services and fibro-scanning were seen as increasing the awareness of the HCV disease among both prisoners and staff (Table 1).

*“Anything that stops us going outside the main gate is good”. (Governor)*

*“The in reach - raises the awareness ...... raises the profile of the disease as a thing”. (Prison officer)*

*“Certainly, the awareness of the Hep C has gone up in the last couple of months with the fibroscanner ... I see more engagement with staff and prisoners”. (Governor)*

**Table 1:** Summary of barriers and enablers to HCV screening and treatment in prisoners

Barriers	Enablers
Increased security needs reduce allocated time for supporting medical services	In-reach medical services reduce requirement for hospital visits
Inadequate staffing and resources	Provision of education and training
Lack of knowledge about HCV among security staff and prisoners	Reassuring prisoners regarding confidentiality
Misinformation regarding historical HCV treatments circulating among prisoners	Opt-out HCV screening on committal
Concerns regarding medical confidentiality	Utilisation of ‘down-time’ in the prison timetable
Prisoners’ fear of treatment side effects and liver biopsy	Prison peer workers
Prisoners’ experience and fear of stigma	On-site hepatology and Fibroscan

## Discussion

This study provides a unique insight into governors’ and prison officers’ views on HCV screening and treatment in Irish prisons. Many of the themes identified in this research including: lack of knowledge, fear of treatment, concerns regarding confidentiality and stigma as barriers to HCV screening and treatment have been reported previously in the literature (Jones et al. 2014; Swan et al. 2010). As outlined above the majority of available qualitative literature published on the topic of HCV in prison is from the prisoners’ and health care staffs’ perspectives (Khaw et al. 2007; Yap et al. 2014). This study identified similar barriers from a security staff perspective and found an unexpected level of knowledge and understanding of this research topic. The focus group narratives demonstrate insight and empathy into the complexity of prisoners’ health needs and illustrates the struggle to provide health care to this group in an environment where prisoner and staff safety and security is paramount.

Participants identified lack of knowledge regarding HCV among prisoners as a concern. Health promotion is a key aspect of prison health care (Møller et al. 2007; Santora et al. 2014). Providing fact sheets on the management of chronic illnesses, including HCV, to prisoners is recommended by the WHO (Møller et al. 2007; WHO 2014). These should be at an appropriate literacy level and in different languages reflecting the demographic makeup of the prison population. Consideration should also be given to the use of video for prisoners with poor literacy skills (Møller et al. 2007). This study identified lack of knowledge about BBV among security staff as a concern. A number of studies have identified this previously with one evaluating training packages to address the issue (Dillon and Allwright 2005; Jack et al. 2017; Perrett et al. 2014). An e-learning package on blood-borne viruses (BBV) piloted in a Welsh prison improved staff understanding of BBV and the authors recommended that it be incorporated into prison staff training (Perrett et al. 2014). Improving knowledge on BBV among prison staff has the potential to alleviate fear among staff but also to reduce the stigma experienced by prisoners in relation to identification of their HCV/HIV status and history of IDU (WHO 2014). The importance of educating security staff about health problems affecting prisoners is a fundamental tenet underpinning prison healthcare (Møller et al. 2007). It is also important that all staff receive training on and understand the complex ethical issues associated with prison health care delivery including those linked to HCV screening and treatment (Allen and Aburabi 2016; Kelk 1999; Levy and Larney 2015; WHO 2014).

The issue of stigma experienced by PWID, prisoners and HCV infected people as a barrier to engagement in health services is commonly reported in the literature (Ford and Bressan 2014; Golden et al. 2006; Swan et al. 2010; Yap et al. 2014). HCV infected prisoners are some of the most marginalised people and their fear and experience of stigma impacts negatively on their engagement with medical services (Fazel and Baillargeon 2011). Stigma that is experienced is closely linked with concerns regarding confidentiality. Security staff in Irish prisons identified this concern as a major block to prisoner engagement in HCV care and provided insights into the complexity of the issue. Maintaining medical confidentiality in prisons is problematic and can often be broken by the types of services that prisoners engage with while incarcerated (Allen and Aburabi 2016). Prisoners attending OST services will be identified as drug users and those attending HIV/HCV in-reach services will have their BBV status declared. Integrating HCV treatment into the prison primary care system has the potential to reduce stigma and protect medical confidentiality (Read et al. 2017).

This study identified peer workers as an enabler to prisoners engaging in HCV screening and treatment and reducing stigma. The use of peer workers in community HCV management is well documented (Arain et al. 2016; Harris and Rhodes 2013; Rhodes et al. 2013; Roose et al. 2014; Treloar et al. 2014). Peer workers can dispel the myths and fears associated with HCV treatment, reduce stigma, enhance mutual trust, enhance social support, and increase knowledge and engagement in HCV care. Evidence supports the effectiveness of prison peer workers at reducing risky behaviours, their acceptability within the prison environment and their positive impact on those that engage with the service (Bagnall et al. 2015; Broadhead et al. 2002; European Centre for Disease Prevention and Control 2018; Macarthur et al. 2016; South et al. 2014).

Prison settings are charcterised by high rates HCV infection and risk behaviours, all of which can potentially impact on staff safety (Larney et al. 2013; Vescio et al. 2008). Many prison officer participants reported feeling concerned about their risk of work-related acquisition of BBV. This concern was heightened by a growing culture of gangland violence in Irish prisons, heightening prisoner related violent behavior whilst incarcerated (Irish Penal Reform Trust 2018).

Implementing evidenced- based harm reduction measures such as needle and syringe programs (NSP), opioid substitution treatment (OST) and vaccination against hepatitis B virus (HBV) can reduce BBV transmission in prisons (Arain et al. 2014). Combined with active case finding and treatment of HCV infection, it has the potential to eliminate HCV infection in prison settings thus making this environment safer for both prisoners and staff (Hajarizadeh et al. 2017; Martin et al. 2015; World Health Organization 2015). Micro-elimination of HCV infection has been reported in some prisons and is increasingly being recommended as an effective HCV public health strategy (Bartlett et al. 2018; Cuadrado et al. 2018; Lazarus et al. 2017; Redman and Sterling 2018). It has the added effect of making the work environment safer for security staff and may impact positively on their engagement with public health strategies aimed and HCV micro-elimination at an institutional level.

This study identifies the need for a structured and systematic approach to HCV screening in Irish prisons. Opt-out screening on committal has previously been shown to increase HCV care uptake in prisons and reduce stigma (European Centre for Disease Prevention and Control 2018; Larney et al. 2014; Morris et al. 2017; Rumble et al. 2015). However concerns have been expressed about the ethical issues that may arise with informed consent and the potential for coercion (Levy and Larney 2015). Committal to prison is identified as stressful for prisoners and HCV screening and treatment is just one of many competing priorities at this time (Levy and Larney 2015; Morris et al. 2017; Rumble et al. 2015). This study identified that “down times” occurring in prison life might provide an opportunity to engage prisoners that had refused opt-out HCV screening at committal.

The use of focus group methodology allowed for a large number of prison officers and governors to engage with the study. It is a practical and acceptable way to conduct research in real life prison settings. The interaction among participants during the group provided the opportunity for an in-depth exploration of topics. A further strength of this study is the national coverage of the governor focus group and the engagement of prison officers from both male and female prison locations. Having prison officer representation from only two out of the 15 prison locations is a limitation of the study. Researcher 1 (DC) was known to most focus group participants which may have impacted on their willingness to fully disclose their views in a positive way. The low number of female participants was also seen as a limitation. The recruitment by invite for the prison officer focus groups may have biased the findings since only those with an interest in the area of health care may have participated. Demographics such as age, gender and length of time in service were not collected since these were deemed to be too sensitive to collect in a work environment and so we cannot determine that focus group participants were representative of the study population.

## Conclusion

Prisoners are from marginalised groups who are underserved by traditional healthcare systems. Their time in prison may offer a unique opportunity for the diagnosis, assessment and treatment of HCV infection. Adapting a whole prison approach to HCV screening and treatment is essential to optimise uptake of HCV care. While the maintenance of prison security is the primary function of prison officers and governors, supporting prison health care is an important role. Providing education around HCV screening and treatment to both prison staff and prisoners has the potential to increase knowledge and reduce fear and stigma. Adopting opt-out screening at committal is supported by both prison officers and governors and is an evidence-based approach to increasing uptake of HCV care among prison populations. The use of peer workers in prison settings is also supported by Irish prison security staff but its implementation needs to be underpinned by a robust management and governance to ensure prison security is not jeopardised. Understanding health care delivery and concerns from a security staff perspective is crucial to optimising the benefits of prison health care. Working jointly towards HCV elimination in prison can have a positive impact both on prisoner health and the prison work environment.

## Abbreviations

BBV: Blood borne virus
HCV: Hepatitis C Virus
HIV: Human immunodeficiency virus
IDU: Injecting drug use
IPS: Irish Prison Service
OST: Opioid substitution treatment
PIS: Participant information sheet
PWID: People who inject drugs
ROI: Republic of Ireland
WHO: World Health Organisation
